# Clinical efficacy of surgery for patients with Chiari malformation type I with syringomyelia: posterior fossa decompression versus posterior fossa decompression with resection of tonsils

**DOI:** 10.3389/fneur.2025.1556026

**Published:** 2025-03-03

**Authors:** Lei Zhang, Ben Li Li, Shuo Wei, Hong Wei Hu, Hong Fu Chen, Yue Chao Fan, Hui Zhang, Pei Zhi Ji

**Affiliations:** ^1^Department of Neurosurgery, Affiliated Hospital of Xuzhou Medical University, Xuzhou, China; ^2^Department of Neurobiology, Xuzhou Medical University, Xuzhou, China

**Keywords:** Chiari malformation type I, posterior fossa decompression, posterior fossa decompression with resection of tonsils, syringomyelia, cerebellar tonsils

## Abstract

**Background:**

The optimal surgical approach for treating Chiari malformation type I (CM-I) with syringomyelia remains a topic of debate. Key areas of controversy include the extent of decompressive craniectomy, the necessity of subarachnoid exploration, and whether to excise the herniated tonsils. In this study, we present our perspectives on these contentious issues through a retrospective analysis of the clinical efficacy of posterior fossa decompression with resection of tonsils (PFDRT) compared to posterior fossa decompression (PFD).

**Methods:**

We conducted a retrospective analysis of clinical data from 162 patients diagnosed with CM-I and syringomyelia who underwent surgical intervention at the Affiliated Hospital of Xuzhou Medical University between January 2017 and December 2022. Among these, 58 patients underwent PFD, while 104 received PFDRT. The efficacy of the treatments was evaluated using the Chicago Chiari Deformity Prognosis Scale (CCOS) at 6 months post-surgery, with scores ranging from 13 to 16 indicating a favorable prognosis. Furthermore, the improvement of syringomyelia was assessed through magnetic resonance imaging (MRI) at the six-month follow-up.

**Results:**

Six months post-surgery, according to the Chiari Clinical Outcome Scale (CCOS) score, the improved rates for the PFD and PFDRT groups were 56.9 and 78.8%, respectively. Additionally, the recovery rates for syringomyelia in these groups were 55.2 and 76%, respectively. Statistically significant differences were observed in both the rates of favorable prognosis and syringomyelic improvement between the two groups (*p* < 0.05). The incidence of complications, including fever, cerebrospinal fluid leakage, intracranial infection, and incision infection, did not differ significantly between the groups (*p* > 0.05).

**Conclusion:**

Our findings indicate that PFDRT yields superior outcomes in syringomyelia improvement and favorable prognoses compared to PFD, while maintaining comparable postoperative complication rates.

## 1 Introduction

Chiari malformation (CM) is a congenital craniocervical junction malformation that is typically characterized by hernia of the cerebellar tonsils through the foramen magnum (1, 2). It is often accompanied by the obstruction of cerebrospinal fluid circulation, thus resulting in syringomyelia or hydrocephalus. There are four types of CM, and Chiari malformation type I (CM-I) accounts for approximately 90% of all CM cases (3, 4). Furthermore, 30–70% of CM-I cases are complicated with syringomyelia. The diagnostic criterion for CM-I is that the herniated cerebellar tonsils are at least 5 mm below the plane of the foramen magnum on cervical spine sagittal magnetic resonance imaging (MRI) (5–9). At present, there is still no consensus regarding the optimal surgical method; posterior cranial fossa decompression is most commonly used for treatment (10). Based on this, the clinical effects of PFD and PFDRT in the treatment of CM-I with syringomyelia were compared and analyzed in this paper, and our views were put forward in order to provide valuable references for clinical treatment.

## 2 Data and methods

### 2.1 Clinical data

This study included 162 patients (85 males and 77 females) with ages ranging from 20 to 65 years and a mean age of 32.9 years (Table 1). The duration of the disease ranged from 4 to 24 months, with an average of 13 months. Clinical manifestations: One hundred and forty-nine patients had irritated nerve symptoms, such as headache and neck and shoulder pain. Limb numbness was observed in 112. Syringomyelia manifestations, such as dissociative sensory disorders, were observed in 77 patients. Seventeen patient had symptoms of cerebral nerve injury and cerebellar dysfunction, such as hoarseness and unsteady walking. Imaging findings: All patients underwent cervical MRI, and CM-I with syringomyelia was clearly present. The tonsils of the cerebellum were herniated downwards into the foramen magnum of the occipital bone, with a lower herniation depth > 5 mm. Posterior fossa decompression (PFD) was performed in 58 patients, and posterior fossa decompression with resection of the tonsils (PFDRT) was performed in 104 patients.

**Table 1:** Demographic information of PFD and PFDRT.

Demographics	Levels	PFD, *n* (%)	PFDRT, *n* (%)	*p*
Sex	Female	24 (41.4)	53 (51)	0.314
	Male	34 (58.6)	51 (49)	
Dizziness, headache, Neck and Back Pain	Exist	49 (84.5)	90 (86.5)	0.901
	Not exist	9 (15.5)	14 (13.5)	
Numbness	Exist	41 (70.7)	71 (68.3)	0.887
	Not exist	17 (29.3)	33 (31.7)	
Syringomyelia manifestations	Exist	33 (56.9)	44 (42.3)	0.106
	Not exist	25 (43.1)	60 (57.7)	
Gait abnormalities, dysphagia	Exist	5 (8.6)	12 (11.5)	0.754
	Not exist	53 (91.4)	92 (88.5)	
Patient age	Mean ± SD	33.1 ± 9.6	32.9 ± 8.8	0.882

### 2.2 Inclusion criteria

The inclusion criteria were as follows: 1. Confirmed diagnosis of CM-I with syringomyelia; 2. primary surgery for CM-I. The exclusion criteria were as follows: 1. CM-I combined with atlantoaxial dislocation that required intraoperative internal fixation; 2. CM-I combined with basilar invagination; in such cases, compression is mainly caused by the anterior odontoid process, which requires odontoid resection; 3. secondary CM-I caused by hydrocephalus and posterior fossa tumors.

### 2.3 Surgical methods

#### 2.3.1 Posterior fossa decompression

**Figure 1:**
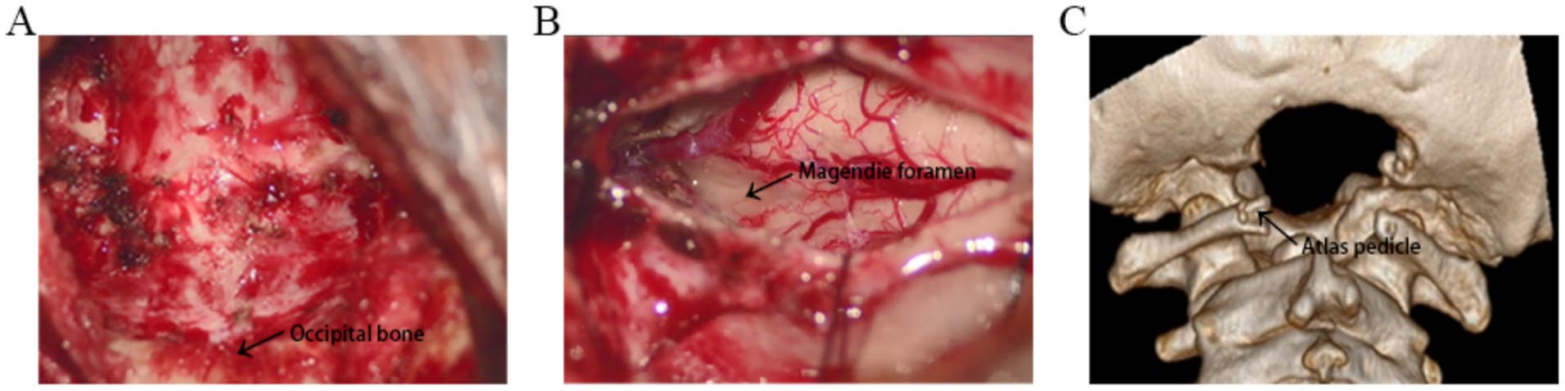
**(A)** The image shows the 3 cm × 2.5 cm craniectomy and removal of the middle part of the posterior arch of the atlas. **(B)** The Magendie foramen was fully exposed, and all the factors affecting the outflow of cerebrospinal fluid from the four ventricles were eliminated. **(C)** Three-dimensional computed tomography reconstruction demonstrates suboccipital craniectomy and removal of the posterior arch of the atlas.

After receiving general anesthesia, the patients were placed in the prone position, the neck was slightly flexed, and the head was fixed with a three-pin head holder. A suboccipital posterior median incision with a length of approximately 4.5 cm was made, the upper margin of the incision was 3 cm above the C2 spinous process, and the lower margin of the incision was 1.5 cm below the C2 spinous process. The skin muscles were incised successively, and the scalp muscles were retracted by a retractor to reveal the posterior arch of the atlas and occipital bone. Approximately 2.5 cm of the middle part of the posterior arch of the atlas was removed, and the occipital bone was removed to form a 2.5× 3 cm bone window (Figure 1A). The upper margin of the bone window exceeded the cerebellar hemisphere-tonsillar notch by 0.5 cm, and approximately 2.5 cm of the posterior margin of the foramen magnum was excised. Suture the muscles, fasciae, and various skin tissues layer by layer.

#### 2.3.2 Posterior fossa decompression with resection of tonsils

After receiving general anesthesia, the patients were placed in the prone position, the neck was slightly flexed, and the head was fixed with a three-pin head holder. A suboccipital posterior median incision with a length of approximately 4.5 cm was made, the upper margin of the incision was 3 cm above the C2 spinous process, and the lower margin of the incision was 1.5 cm below the C2 spinous process. The skin muscles were incised successively, and the scalp muscles were retracted by a retractor to reveal the posterior arch of the atlas and occipital bone. Approximately 2.5 cm of the middle part of the posterior arch of the atlas was removed, and the occipital bone was removed to form a 2.5× 3 cm bone window (Figures 1A,C). The upper margin of the bone window exceeded the cerebellar hemisphere-tonsillar notch by 0.5 cm, and approximately 2.5 cm of the posterior margin of the foramen magnum was excised. A straight or Y-shaped dural incision was made. The enlargement and reconstruction of the cisterna magna are performed, and part of the occipital bone is retained to support the cerebellar hemisphere and prevent cerebellar ptosis. The subarachnoid space was routinely explored during the operation to separate the bilateral cerebellar tonsils, the presence of arachnoid adhesion between the bilateral lower hernia tonsils and between the lower hernia tonsils and the brainstem was investigated. Sharp, separation was performed to reveal the median foramen of the fourth ventricle. The tonsils were gently manipulated using microsurgical instruments. Bipolar coagulation was applied at a setting of 6 watts, focusing on the posterior, medial and inferior surfaces of the tonsils. Approximately 30–40% of the tonsillar tissue was resected, with incisions made along the inferior poles to achieve an average volume reduction of 20–30%. Care was taken to preserve the surrounding neural and vascular structures. All the factors affecting the outflow of cerebrospinal fluid from the four ventricles were eliminated, including the obstruction of the foramen magnum by cerebellar tonsillar herniation, the covering of the spinal central canal by the cerebellar tonsils, the presence of arachnoid adhesion between the cerebellar tonsils, the presence of arachnoid adhesion between the cerebellar tonsils and the brainstem, and the presence of arachnoid adhesion and cysts in the cisternal magnum. During the operation, the Magendie foramen was fully exposed to ensure the flow of cerebrospinal fluid (Figure 1B). The dura mater was sutured with an artificial dural patch. Watertight closure was achieved. The closure was reinforced with bioprotein glue. The cisterna occipitalis was reconstructed after dural enlargement. Suture the muscles, fasciae, and various skin tissues layer by layer. Beecher et al. (11) offer further context and support for our surgical methodology.

### 2.4 Follow-up and evaluation of surgical outcomes

**Figure 2:**
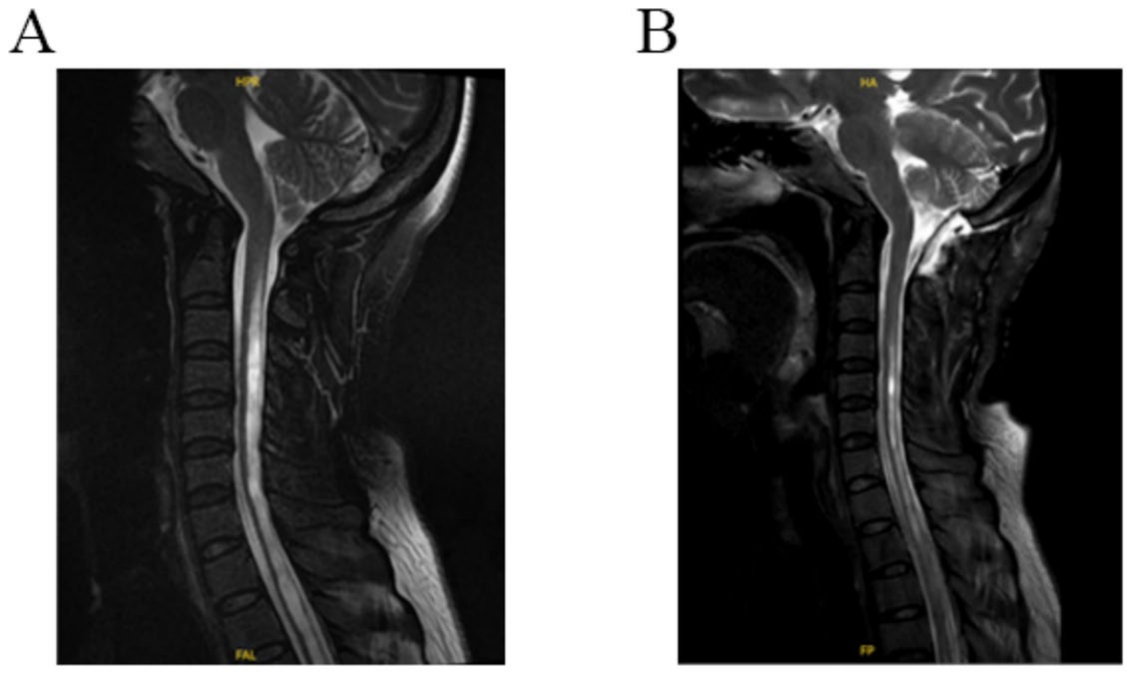
**(A)** Preoperative T2 MR-weighted images showing cerebellar tonsils through the foramen magnum and syringomyelia. **(B)** Postoperative T2 MR-weighted 3-day image revealing a decrease in the size of syringomyelia and an enlargement of the subarachnoid space of the foramen magnum.

All patients were followed up for 6 to 33 months, and the symptoms and signs before and after surgery were compared. Six months after surgery, the outcome was evaluated according to the Chicago Chiari outcome scale (CCOS) scores: 13–16 were improved group; 9–12 were unchanged group, and 4–8 was worse group (12, 13). Changes in syringomyelia were observed on MRI of the cervical spine, which was more than 50% smaller than that before surgery and were judged to be improved (Figure 2). Postoperative complications such as fever, incision and intracranial infection and cerebrospinal fluid leakage were observed.

### 2.5 Statistical method

SPSS 26.0 software was used for analysis. Counting data *χ*^2^ Test or Fisher exact probability test; those data with normal distribution were expressed as the Mean ± SD by *t*-test of two independent samples; *p* < 0.05 was considered to be statistically significant.

## 3 Results

### 3.1 The symptoms and signs of CM-I improved

Six months after operation, according to the Chiari Clinical Outcome Scale (CCOS) score, the improved rates for the PFD group and PFDRT group was 56.9 and 78.8%, respectively. Changes in syringomyelia were observed on MRI of the cervical spine. The recovery rates of syringomyelia were 55.2 and 76%, respectively. There were statistically significant differences in the rate of good prognosis and syringomyelic improvement between the two groups (*p* < 0.05; Table 2).

**Table 2:** Comparison of surgical treatment results between the two groups.

Outcomes	Levels	PFD, *n* (%)	PFDRT, *n* (%)	*p*
CCOS	Unimproved	25 (43.1)	22 (21.2)	0.006
	Improved	33 (56.9)	82 (78.8)	
Syringomyelia	Better	32 (55.2)	79 (76)	0.011
	Not better	26 (44.8)	25 (24)	
Fever	Exist	15 (25.9)	32 (30.8)	0.632
	Not exist	43 (74.1)	72 (69.2)	
CSF fluid	Exist	0 (0)	6 (5.8)	0.153
	Not exist	58 (100)	98 (94.2)	
Intracranial infection	Exist	0 (0)	5 (4.8)	0.222
	Not exist	58 (100)	99 (95.2)	
Infection of incisional wound	Exist	2 (3.4)	5 (4.8)	0.996
	Not exist	56 (96.6)	99 (95.2)	
Surgery times	Mean ± SD	90.7 ± 9.3	121.5 ± 18.0	<0.001
Hospital days	Mean ± SD	12.2 ± 1.8	13.1 ± 2.7	0.009
Bleeding volumes	Mean ± SD	80.7 ± 9.3	127.8 ± 19.0	<0.001

### 3.2 Compared with the PFDRT group

The operative time and intraoperative blood loss were significantly reduced in the PFD group (*p* < 0.05), and the postoperative hospital stay was shortened (*p* < 0.05). There was no significant difference in the incidence of postoperative complications (fever, intracranial infection, incision infection and cerebrospinal fluid leakage) between the two groups (*p* > 0.05; Table 2). All of these complications were cured after active treatment.

## 4 Discussion

CM is a congenital malformation in which the cerebellar tonsils herniate downwards into the foramen magnum, and it is often accompanied by syringomyelia. The pathogenesis of CM has yet to be fully elucidated. Bone structural abnormalities, including abnormal development of the occipital bone and shortened slope length may play an important role in the etiology of CM. Previous studies have confirmed that the length of the slope in patients with CM without basilar invagination is significantly shorter than that in normal people, and the shortened slope can be used as an auxiliary diagnostic basis for CM (14). Abnormal development of the occipital bone may be another important cause of CM (15). The inclination angle of the occipital bone near the foramen magnum area of the CM was significantly increased, which provided a basis for the rationality of decompression in the foramen magnum area, especially in the treatment of CM with small bone window decompression (16).

An abnormal bone in the foramen magnum results in a reduced volume of the posterior cranial fossa and congestion of the posterior cranial fossa, resulting in deformed and ectopic cerebellar tonsils and impaction of the lower hernia cerebellar tonsils in the foramen magnum. CSF may first cause obstruction of the cerebrospinal fluid behind the cerebellar tonsils, followed by obstruction of the cerebrospinal fluid between the slope and the ventral side of the brainstem. The normal pulsating movement of cerebrospinal fluid through the foramen magnum is blocked, resulting in syringomyelia. The symptoms of CM are very complex. Due to the cerebellar tonsillar hernia, the cranial nerves and cervical nerve roots are compressed, resulting in hoarseness, dysphagia, neck pain and limited movement. Due to the compression of the medulla oblongata and the upper cervical cord, limb movement and sensory disorders may occur. In the case of syringomyelia, sensory separation or muscle atrophy of both upper limbs may occur. When the cerebellum is involved, ataxia, walking instability and nystagmus may occur. Symptoms of hydrocephalus and increased intracranial pressure can occur when cerebrospinal fluid circulation is blocked (17).

There is still no consensus on the choice of surgical method for treating Chiari malformation, and there are many surgical methods available at present. Common surgical procedures include PFD, PFDD, and PFDRT. Durham and Fjeld-Olenec concluded that PFD without dural incisions had the same improvement effect on the clinical symptoms of patients with CM-I as PFDD (18). It has been reported that duraplasty is superior to PFD (19, 20). It has been reported in the literature that PFDRT is significantly better than the other two surgical methods for improving symptoms and syringomyelia in CM-I patients (21–23). Nevertheless, due to the potential for increased postoperative complications associated with PFDRT, certain surgeons remain hesitant to apply this procedure in patients with Chiari Malformation Type I (CM-I).

At present, there is also controversy regarding whether to remove the cerebellar tonsils. Some researched have suggested that it is difficult for PFD to achieve therapeutic effects (24). In patients with CM-I, posterior fossa small bone window decompression followed by subdural decompression or subarachnoid decompression can effectively improve CSF flow obstruction and increase the posterior fossa volume (25). Stanko et al. suggested that resection of the cerebellar tonsils could significantly improve syringomyelia in patients with CM-I compared with that in patients with PFDD (26). Some scholars believe that removal of the cerebellar tonsils should not be performed routinely. The arachnoid was kept intact to avoid damage to the central nerve and blood entering the subarachnoid space, causing adhesion. Postoperative headache, dizziness and aseptic meningitis are more common in these patients (27). In particular, the patient was complicated with syringomyelia, indicating a disturbance of cerebrospinal fluid circulation in the foramen magnum. It has been reported that the factors causing cerebrospinal fluid disturbance in the foramen magnum are as follows: cerebellar tonsillar hernia blocking foramen magnum and covering foramen Magendie in 88% (44/50), arachnoid adhesion between cerebellar tonsils in 36% (18/50), and arachnoid adhesion between cerebellar tonsils and brainstem in 18% (9/50) of cases (28).

This paper compared the efficacy of PFD and PFDRT in the treatment of CM-I complicated with syringomyelia from the following aspects, including good prognosis rate, syringomyelia improvement rate and postoperative complications. In our study, according to the Chiari Clinical Outcome Scale (CCOS) score, the improved rates for the PFD group and PFDRT group was 56.9 and 78.8%, and the recovery rate of syringomyelia was 55.2 and 76%, respectively. There were statistically significant differences in the rate of good prognosis and syringomyelic improvement between the two groups (*p* < 0.05). At the same time, compared with PFD group, the incidence of postoperative complications such as fever (*p* = 0.632), cerebrospinal fluid leakage (*p* = 0.153), intracranial infection (*p* = 0.222) and incision infection (*p* = 0.996) in PFDRT group was not statistically significant (*p* > 0.05). In some patients, it is recommended that cerebrospinal fluid (CSF) dynamic imaging and intraoperative ultrasound be utilized, as suggested by Fan et al. (29). Our observations suggest that the lower hernia of the cerebellar tonsils influences the obstruction of cerebrospinal fluid circulation. Consequently, we propose that subarachnoid surgical interventions primarily involve electrocoagulation or partial resection of the cerebellar tonsils, along with exploration of the median foramen of the fourth ventricle. This approach aims to achieve anatomical relief of the lower herniation malformation of the cerebellar tonsils. Our findings align with previous research, which indicates that a higher rate of spinal cavity relief can be attained through the electrocoagulation of the cerebellar tonsils, thereby facilitating their reduction and alleviating obstruction and compression at the central canal opening of the spinal cord (30). Therefore, we believe that it is necessary to conduct subarachnoid exploration and excision of the tonsils in patients with syringomyelia. Compared with the PFD group, the operative time, intraoperative blood loss and hospital stay in the PFDRT group were statistically significant (*p* < 0.05). However, the actual differences were not significant. We believe that strengthening surgical proficiency and enhancing postoperative management may further reduce this gap.

There are also different approaches for bone window decompression. Some scholars believe that posterior cranial fossa decompression should be sufficient, that the bone window range should be large enough, and that even an occipital bone resection of 5 cm × 4 cm should be achieved. When the cerebellar tonsillar has herniated to the level of the atlas, PFD is likely insufficient to restore cerebrospinal fluid circulation. They suggested that resection of the posterior arch of the atlas would facilitate reconstruction of the cisterna and duraplasty (31). However, excessive occipital resection can lead to cerebellar ptosis and symptom aggravation. In our experience, the median portion of the posterior atlas arch is approximately 2.5 cm long, the width of the posterior margin of the foramen magnum is equivalent to the width of the posterior atlas arch removed, and the upper boundary of the bone window and the external turning point (the external upper corner) are more than 5 mm longer than the width of the cerebellar hemisphere-tonsillar notch of the dura. This can ensure adequate decompression, fully expose the cerebellar tonsils, remove the tonsils when necessary, expand and rebuild the cisterna occipitalis, and prevent cerebellar ptosis.

One interesting thing we found in our study was that the patient profile emerging from Table 1 differs from the one frequently described in the English literature. For example, the female to male ratio in American and European patients is 3:1 (32–38). In another study, 55 % of the patients were female (39). Most cohorts reported a female predominance in CM, with the proportion of female to male patients varying across studies. This sex disparity is observed in both pediatric and adult populations. The underlying cause of this difference remains unclear. This discrepancy may reflect anatomical variations, such as the flat occiput and supraocciput commonly seen in Far East populations, which could influence the pathophysiology and presentation of this condition. We suspect that regional differences or economic differences may also affect the results.

In this research, the determination of surgical methods was primarily grounded in the patients’ comprehensive conditions. This included the severity of their symptoms, the extent of cerebellar tonsillar herniation, and the degree of syringomyelia. For cases exhibiting more pronounced severity in the aspects mentioned above, we tended to adopt the PFDRT surgical approach. Simultaneously, clinicians carefully considered the preferences of both the patients and their families. They would then integrate their extensive clinical experience and professional expertise to reach the final decision. Additionally, we meticulously analyzed the distribution of surgical procedures across different time periods. The analysis revealed no conspicuous tendency of surgical procedures evolving over time within the same institution. We must explicitly state that, despite implementing numerous measures during the study design and data analysis phases to mitigate potential biases, the inherent subjectivity in surgical selection may still influence the research outcomes. Concurrently, we will also deliberate on strategies to further optimize the decision-making process for surgical mode selection in future research endeavors. This optimization aims to enhance the reliability and clinical applicability of the study results. While the 6-month postoperative follow-up period provides valuable initial insights, it is relatively short for evaluating long-term outcomes. We intend to continue monitoring this patient cohort to assess the durability of the observed improvements and to identify any potential delayed complications.

## 5 Conclusion

Although different surgeons perform the operation in different ways, the primary goal of the operation is the same: to relieve the brain stem and nerve compression, restore the normal flow of cerebrospinal fluid in the foramen magnum, and reduce the size of the syringomyelia (40). Our surgical strategy for the treatment of cerebellar subtonsillar hernia malformation type I with syringomyelia is effective and has a low complication rate. Therefore, for patients with syringomyelia, we believe that the PFDRT surgical strategy should be recommended. This study followed the Strengthening the Reporting of Observational Studies in Epidemiology (STROBE) reporting guidelines for observational studies (41). However, as a single-center retrospective study with a small sample size, further research with a larger sample, multi-center involvement, and prospective design is needed to confirm these findings.

## Data Availability

The raw data supporting the conclusions of this article will be made available by the authors, without undue reservation.
